# Practical Antisepsis in Surgery1Presented at the Thirty-seventh Annual Meeting of the American Veterinary Medical Association, Detroit, September, 1900.

**Published:** 1900-10

**Authors:** G. A. Johnson

**Affiliations:** Sioux City, Iowa


### PRACTICAL ANTISEPSIS IN SURGERY.[1]

By G. A. Johnson, D.V.M.,
SIOUX CITY, IOWA.

During the last quarter of the nineteenth century surgery has made such rapid progress that now major and difficult operations are taken as matters of fact and have ceased to be the wonder of the times; especially is this true in surgery of mankind.

This marvellous progress is very largely due to a better knowledge of the röle played by microörganisms in the process of wound repair. Medical literature is replete with treatises ranging from the short essay to the voluminous text-book upon the subject of antisepsis in its various forms. While the surgeons of the human school have succeeded in gaining a position nearer that of perfection than have those of the veterinary school, it is not to be understood that veterinary surgery has not made great progress.

We have naught but commendation for the veterinary surgeon, who, being desirous of advancing his profession and establishing a reputation for himself by attempting to follow the methods and modus operandi of the eminent surgeons of mankind, and counselling his colleagues to go and do likewise, nor is it strange that in so doing he should at times have been carried beyond the essential landmarks of his profession by the swirl of "the pace that kills"

[1] Presented at the Thirty-seventh Annual Meeting of the American Veterinary Medical Association, Detroit, September, 1900.

in attempting to get similar results where conditions are so materially different as they are between the human and the brute patient.

From a clinical view-point we may consider wounds as surgical or accidental ; surgical wounds are such as result from surgical operations—all others as accidental.

While it is not within the province of this paper to enter into a discussion of the physiological or pathological phenomena of the process of repair, a short discussion of the classification of these phenomena may not be out of place.

From a physiological view-point there is no material difference in the phenomena that takes place when a wound is immediately closed and normal relations are resumed in a few days, without the formation of pus, and in one that remains open and pus is formed. The difference is one of degree rather than of kind.

From a pathological view-point there are two classes of wounds—aseptic and infected. Surgeons of the human school are quite generally agreed to use the pathological classification whereby all wounds are considered to heal by primary or secondary intention—that is, all septic wounds are said to heal by first intention, primary or immediate union, no matter how much time may be required for the completion of the process of repairs, provided it is accomplished without the formation of pus, while all infected wounds—that is, wounds where the process of repair is accompanied by the formation of pus—are said to heal by secondary intention or granulation. But from a clinical view-point, are not veterinarians justified in dividing Class 1 into two subdvisions, thereby having three classes of wound repair ? Thus, in Class 1 we would have healing by first intention, primary or immediate union, where the aseptic parts are brought into close apposition and the normal functions are resumed in a few days. In Class 2, or healing by secondary intention, where the parts of an aseptic wound cannot be brought or kept in close coaptation, but where the process of repair is carried to completion without the formation of pus; and Class 3, or union by granulation, where the process of repair is accompanied by the formation of pus. Thus, we would have two classes of repair in aseptic wounds and one class in infected wounds.

While this classification is purely an arbitrary one, I believe its retention will give lucidity, hence its use in this paper.

It should be the desire of every surgeon to have all of his cases recover as rapidly and with as little inconvenience to his patient or its owner as possible; that is, to have all of his operations terminate by primary union when possible.

This being the pinnacle of perfection, let us briefly consider the conditions that are necessary for its success. Healing by primary or immediate union depends upon three conditions, namely : asepsis, coaptation of the parts, and physiological rest. The healing of a wound by primary union might be likened to the erection of an edifice upon a tripod. If one or more of the legs of the tripod are broken, down comes the structure. Asepsis, coaptation, and physiological rest form the tripod that sustains healing by immediate union, and the least defect in either of them renders such a union an impossibility.

The conditions necessary for healing by secondary intention is that the wound be kept aseptic.

The surgeon of the human school can usually acquire an aseptic wound through the use of a disinfected operating-room, sterilized instruments and dressings, close attention to the disinfection of the person of the patient, the clothing, bedding, etc., and of himself and his assistants ; by means of position, ligation, torsion, and wiping he can control hemorrhage, free the wound of blood-clots and serum ; and with sutures buried and otherwise he can usually coapt the parts, uniting muscle to muscle, tendon to tendon, and bone to bone ; and through the submissive and usually assisting disposition of the patient, supported by bandages, splints, etc., he can generally secure the necessary physiological rest.

Veterinary practice presents a field strikingly in contrast to that of the surgeon of mankind. The veterinary surgeon may be so fortunately situated as to have all of the accessories to his work as favorable to good results as the surgeon in human practice, but when it comes to the patient, instead of having a usually submissive and assisting subject, he has a defensive combatant from start to finish. But to the ordinary veterinary surgeon, with his patient cast on the windy side of a barn upon some old musty hay or straw, or, as more frequently is the case, on the bare ground, which is either muddy or dusty, with no opportunity for properly disinfecting the body of his patient, with dirty assistants and the gentle zephyrs scattering germ-laden particles of dust in all directions, an aseptic wound means much.

But let us presume that he has succeeded in performing an aseptic operation, the impossibility of securing and maintaining coaptation of the parts, in most wounds, precludes healing by first intention; while, on the other hand, if he has succeeded in getting good apposition of the parts of an aseptic wound, can he control the patient so as to secure physiological rest except in a few special regions ?

Accidental wounds are usually infected, and it is only by strict observance of the principles of antisepsis that surgical wounds are kept aseptic.

In operating I usually follow as closely as circumstances will permit the following lines :

1. Proper dieting of the patient.

2. Cleansing and disinfecting the field of operation, (*a*) removal of the hair ; (*b*) washing the parts with soap and water ; (*c*) washing with a disinfectant. For this purpose I prefer crude (full strength) creolin well rubbed in over the field of operation, and allowed to remain from three to five minutes and then washed off. If allowed to remain too long it will produce too much irritation, resulting in œdema of the parts. After the first application of the creolin has been washed off, I usually apply a very small quantity of creolin to the wet surface, and allow this to remain until I am ready to operate.

3. Such sterilized instruments as are likely to be needed during the operation are placed in a tray containing a 1 per cent. solution of formalin.

4. (*a*) Confining the animal by casting or otherwise ; (*b*) giving the parts a final washing with sterilized water or a weak solution of creolin. I usually apply a very light coating of creolin over quite a surface surrounding the field of operation, with a view of preventing hands or instruments from becoming infected by accidentally coming in contact with these parts.

5. Disinfection of the hands. Usually I rub my hands with some crude creolin, then wash them, seeing that the nails are well cleaned, then wash with a 1 per cent. solution of formalin.

6. The operation, including hæmostasis.

7. Dressing the wound. Remove all blood-clots, shreds of tissue, and stop seeping as much as possible by gently wiping with pledgets of cotton or gauze. The further dressing depends very largely upon the location or character of the wound. If the wound is such that the parts can be brought into close apposition and retained thus, I put into the cavity a limited quantity of some non-irritating, prohibitive antiseptic, as boric acid or iodoform, that will not interfere with immediate union, but that will have a tendency to arrest the development of any germs that may have accidentally found lodgement in the wound. Then fix the parts by means of aseptic sutures, cover the parts with some of the same or similar powder as used in the wound, and over this apply a quantity of gauze or absorbent cotton, upon which has been applied a goodly quantity of a similar powder, and retain this in place with bandages.

If the wound is such that the parts cannot be brought into apposition or be kept so, but where the edges of the skin may be brought together, fill the cavity thus formed with a non-irritating antiseptic powder and suture the skin, and if practical apply a protective dressing of gauze and bandages. If the gauze and bandages cannot be applied, a coating of antiseptic collodion or wound gelatin may be applied over the wound. Remove the dressing and sutures as soon as there is any evidence of the sutures giving way or that the wound has become infected; cleanse the wound and repeat the treatment as at first.

To illustrate, I will cite the treatment of a case of scirrhous cord in a horse. The horse, having been dieted for a couple of days, was cast and operated upon under chloroform anæsthesia with antiseptic precautions as above outlined. An egg-shaped tumor about six inches long by four inches in diameter was removed, leaving a cavity so large as to preclude coaptation of the parts. The wound was cleansed of blood-clots, shreds, etc., and seeping partially checked by means of wiping with gauze, and a powder composed of about three drachms each of boric acid and acetanilid was put into the cavity, the wound in the skin closed with sutures, and the parts well dusted with some of the same powder as used in the wound. The animal was allowed to get up, and was placed in a box-stall in a large boarding barn, and kept on a light diet for a few days. The case made a rapid and complete recovery, and was driven on the tenth day after the operation.

Following the operation there were no fever, loss of appetite, or swelling of the parts, other than a slight œdema of the sheath; no pus was formed, except some around the sutures about a week after the operation, notwithstanding the fact that on the second day after operating I introduced my finger between the sutures, breaking down the adhesions to allow the escape of a small quantity of serum that had collected in the cavity. The only other treatment used was the daily application of cold water from a hose attached to a city hydrant.

The same modus operandi, except the dieting, was followed in the castration of a cryptorchid and in the removal of a tumor from the breast of a horse. The tumor case made a rapid recovery, leaving but a slight scar, but I never heard from the castration case after the operation.

In wounds where there is a large cavity, but where the powder and sutures cannot be applied with a reasonable assurance of success, pack the cavity with antiseptic gauze or absorbent cotton. I prefer the gauze, as it is easier to introduce ; it is retained better, and can be withdrawn with less trouble. The desired amount of liquid or powdered antiseptic can be applied to, or within the folds of, the gauze. This packing should be changed as frequently as the case demands, usually once a day or every second day. I have treated two cases of fistula of the withers and one abscess of the shoulder by this method with good results.

In wounds where a large quantity of skin has been destroyed, disinfect the wound and dust freely with some desiccating antiseptic powder frequently enough to secure healing under a dry crust or scab. For this purpose Dr. Frick recommends a powder composed of iodoform and pulverized sugar, equal parts.

In the treatment of open joint the opening should not be allowed to close until the interior of the joint has been rendered aseptic. This may be accomplished in ordinary cases, where the destructive process has not advanced sufficiently to destroy the articular cartilage, by thoroughly washing out the joint with proper antiseptics and protecting the external wound with a powdered antiseptic, reinforced by a dressing of gauze and bandages when practical. For this class of cases I prefer a corrosive sublimate solution (1 to 1000 to 5000 parts). If the wound cannot be thoroughly cleansed through the original opening, make one or more other openings that will admit of this being accomplished. Other accessory treatment, such as internal medication, splints for immobilizing the parts, slings, etc., should be used according to the indications of each case and the temperament of the patient.

In treating infected wounds the first step should be to thoroughly wash the parts (wound and surrounding region) with soap and water and cleanse with an antiseptic solution, then by means of a knife, scissors, or curette remove all foreign bodies, shreds, necrosed or lacerated tissues, exuberant granulations, etc. Then wash the wound with an antiseptic solution until the contaminating germs are destroyed. Then treat according to the indications of the case ; in other words, render the septic wound aseptic and treat accordingly. I am of the opinion that we frequently fail in our attempts to cleanse infected wounds because we do not apply active germicidal remedies for a sufficient length of time to destroy all germ life. But better results will follow the application of a solution of medium strength for a longer time than of a stronger solution for a shorter time. On the other hand, it is of very little practical value to render a wound aseptic and permit it to immediately become reinfected.

It is quite generally agreed by veterinary surgeons that it is impracticable if not impossible to obtain sufficient immobility of the parts to secure healing by primary union, except in a few regions, as the lower part of the extremities, the inguinal region, the face, and the ears. But this should not deter us from employing the principles of antiseptics, but we should bend our energies toward making the theory apply to the case, rather than trying to make the case fit the theory. There is no question but that we should attempt to secure union by first intention when and wherever practical. But when this cannot be reasonably expected we should try to secure union by secondary intention—that is, healing by granulation without pus, for the formation of pus indicates a destructive process, and its presence always retards the process of repair.

Judging from results so far obtained, I am led to believe that a majority of wounds that domestic animals are subject to may be healed by secondary union through the use of large quantities of non-irritating antiseptics in the form of a powder or gauze packing. By the use of a sufficient quantity of a preparation that will not interfere with the process of repair, but that will check the development of if it does not destroy pathogenic organisms, we not only prevent the introduction of germs, but also arrest the development of such as may have found lodgement within the wound. As a rule, a dry dressing is preferable to a liquid dressing for wounds that are expected to heal under a scab, because it will form a better protection by remaining upon the surface better and longer.

While surgeons are quite generally agreed that hydrargyri bichloride cor. stands at the head of the list of chemical disinfectants, the selection of the agent used will ever remain largely one of personal choice of the surgeon. Personally I prefer creolin for disinfecting the field of operation, especially in practice outside of a hospital, because of its easy application, not requiring so many vessels to properly carry on the work.

For packing large cavities we need a prohibitive germicide that is cheap, that is light and bulky, that will not irritate the parts, that will not act as a foreign body, that will not dissolve too rapidly, that will not produce unfavorable systemic disturbances, but will prevent germ development. A combination of boric acid and acetanilid will prevent germ development, but it dissolves too rapidly, and has a tendency to stimulate a serous exudate in the wound. Iodoform is too expensive, too heavy, and may produce too great systemic disturbances when used in large quantities. I am of the opinion that a combination of two or more drugs of this class that have different degrees of solubility and systemic action will give better results than any one remedy that is on the market at the present time, although I have not as yet had an opportunity to use glutol (a combination of gelatin and formalin), a remedy that has recently come into use in human practice for packing cavities where the parts cannot be coapted, nor iodide of starch, a preparation that seems to have a number of the essential attributes.

Cheese cloth, a thin gauzy cloth, that can be purchased at any dry goods store for from three to five cents per yard, when thoroughly boiled in a solution of sodium chloride or boric acid, and dried in an oven, will answer all purposes equally as well as the higher-priced medicated or surgical gauzes on the market. For sterilizing instruments and dressings, such as absorbent cotton, gauzes and bandages, nothing is so efficient as moist or dry heat.

The application of accessory treatment, such as bandages, splints, etc , and the use of a sling will depend largely upon the character of the wound and the temperament of the patient.

This paper has been prepared more especially with reference to the treatment of wounds in the larger domestic animals, but the principles of antisepsis apply equally as well to the smaller animals, differing only in the special application.

In conclusion, I wish to call your attention to a most vital point, and that is, that the surgeon should never lose sight of the fact that while a theory or principle is usually broad enough to cover all cases no set rule can be successfully adopted, but that each and every case should be treated according to its special indication.

---

*Bulletin* No. 60 of the Agricultural Experiment Station of Louisiana treats of anthrax and charbon as they specially affect the district of Louisiana, and in the investigations conducted by Dr. W. H. Dalrymple and the State Bacteriologist, W. R. Dodson, many valuable deductions are made. The bulletin closes with the following important paragraph : Relative to preventive vaccine, it is of the utmost importance that it should be fresh and reliable, and that all those handling it, whether dealers in it or stock-owners, should follow carefully the instructions (which generally accompany the material) necessary for its preservation and reliability. If this is not done the vaccine will deteriorate, may become infected, and, in fact, be rendered useless or even dangerous.

